# PredPS: Attention-based graph neural network for predicting stability of compounds in human plasma

**DOI:** 10.1016/j.csbj.2023.07.008

**Published:** 2023-07-07

**Authors:** Woo Dae Jang, Jidon Jang, Jin Sook Song, Sunjoo Ahn, Kwang-Seok Oh

**Affiliations:** a Data Convergence Drug Research Center, Korea Research Institute of Chemical Technology, Daejeon 34114, Republic of Korea; b Department of Medicinal and Pharmaceutical Chemistry, University of Science and Technology, Daejeon 34129, Republic of Korea

**Keywords:** Plasma stability, Drug discovery, ADME, Graph neural network, Attention analysis, Machine learning, Artificial intelligence, Pharmacokinetic property

## Abstract

Stability of compounds in the human plasma is crucial for maintaining sufficient systemic drug exposure and considered an essential factor in the early stages of drug discovery and development. The rapid degradation of compounds in the plasma can result in poor in vivo efficacy. Currently, there are no open-source software programs for predicting human plasma stability. In this study, we developed an attention-based graph neural network, PredPS to predict the plasma stability of compounds in human plasma using in-house and open-source datasets. The PredPS outperformed the two machine learning and two deep learning algorithms that were used for comparison indicating its stability-predicting efficiency. PredPS achieved an area under the receiver operating characteristic curve of 90.1%, accuracy of 83.5%, sensitivity of 82.3%, and specificity of 84.6% when evaluated using 5-fold cross-validation. In the early stages of drug discovery, PredPS could be a helpful method for predicting the human plasma stability of compounds. Saving time and money can be accomplished by adopting an in silico-based plasma stability prediction model at the high-throughput screening stage. The source code for PredPS is available at https://bitbucket.org/krict-ai/predps and the PredPS web server is available at https://predps.netlify.app.

## Graphical Abstract

**Figure:**
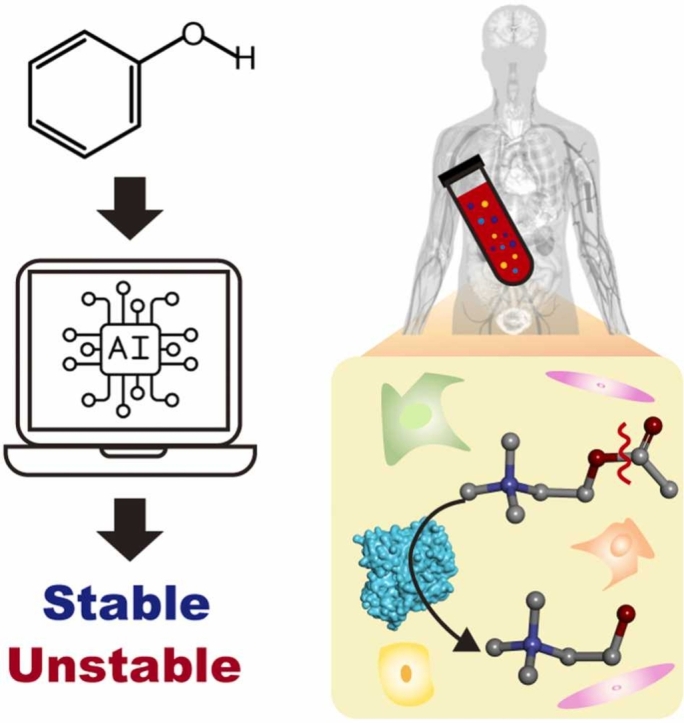

## 1 Introduction

The stability of compounds in the human plasma plays a crucial role in drug discovery and development. Rapidly degraded compounds in plasma tend to have low bioavailability and poor in vivo efficacy. Furthermore, poor plasma stability during sample storage or analysis processes could result in misleading in vivo drug concentrations. Plasma stability is considered an important factor for advanced compounds in drug discovery and development because accurate determination of drug concentration in biological samples is critical for pharmacodynamics–pharmacokinetic studies in preclinical and clinical practice [1], [2]. In addition, the plasma stability profiles of compounds could alert drug discovery teams to modify the molecular structure to improve physicochemical properties and help prioritize molecules for subsequent development [3]. Therefore, plasma stability assays should be conducted early in the drug development process to reach optimal therapeutic concentrations in the clinical phase. However, evaluating plasma stability for large chemical libraries through in vitro or in vivo assays is challenging due to high costs, time requirements, and labor intensity. These limitations highlight the need for an in silico plasma stability prediction tool for quick examination of numerous compounds in the early drug development stages.

In the human body, most drugs are chemically converted via liver metabolism. Computer-based techniques for predicting metabolic stability in human liver microsomes have been developed to assess chemical stability [4], [5]. In addition to liver metabolism, compound decomposition can be catalyzed in the plasma by multiple enzymes, such as hydrolases and esterases. The stability of liver microsomes may differ from that of plasma because plasma and microsomal enzymes are dissimilar. Blood contains several hydrolytic enzymes, such as cholinesterase, aldolase, lipase, dehydropeptidase, alkaline, and acid phosphatase [6], [7]. Plasma degradation is possible if the compound has an affinity for one of these plasma enzymes and a hydrolysable group at the proper position. Certain classes of drug molecules, such as those containing esters, amides, lactones, lactams, carbamides, sulfonamides, and peptic mimetics, are prone to enzymatic hydrolysis by plasma esterases, amidases, or proteases [1]. In this study, plasma stability refers to the ability of small molecules to resist enzymatic degradation by plasma enzymes. This degradation process can convert active drug molecules into inactive or less active metabolites. Therefore, high plasma stability indicates that a small molecule can maintain its structure and function in the presence of plasma enzymes, ensuring that the drug remains active and can reach its intended target site in the body to exert its therapeutic effect. Conversely, low plasma stability suggests that the drug is rapidly metabolized in the bloodstream, which could potentially decrease its effectiveness.

Serum and plasma are the liquid parts of blood that are widely used in drug discovery research. Serum is the liquid that remains after blood clots, and plasma is the liquid that contains coagulation factors such as fibrinogen by adding an anticoagulant. Since these differences could influence the research outcomes, it is necessary to select the appropriate one for the intended purpose. This study focused on developing a model to predict compound stability in human plasma.

There are several factors that make the prediction of plasma stability difficult. Species differences in the distribution and activity of plasma enzymes can lead to differences in the plasma stability profiles of animal species [8], [9]. In addition, plasma stability is difficult to predict because it is greatly affected by the surrounding atoms, such as steric hindrance and electron-withdrawing groups [10], [11]. To date, only a few chemical functional groups are known for their plasma stability. Therefore, it is necessary to develop a computational tool based on deep learning that is trained based on the local and global information of a compound to predict plasma stability in human plasma.

Plasma stability can be used to profile prodrugs where rapid conversion in plasma is desirable. Medicinal chemists can take advantage of plasma reactions as a part of a prodrug approach. The prodrugs improve permeability and oral bioavailability so that high concentrations of the prodrug reach the bloodstream. Hydrolytic enzymes cleave the prodrug to release the active drug in the blood. For instance, the newly approved antiviral drug for COVID-19, remdesivir, adopts a unique strategy for the delivery of phosphate prodrugs. It is administered intravenously to reduce payload release during transit, thereby optimizing payload dispersion within the tissues [12]. Plasma stability prediction tools can be helpful in rapidly designing appropriate prodrugs that are intrinsically unstable in the plasma.

Machine learning (ML) and deep learning (DL) have found applications in evaluating molecular properties like absorption, distribution, metabolism, excretion, and toxicity (ADMET) for drug discovery and development [13], [14], [15]. For example, ML and DL models that predict molecular properties, such as blood-brain barrier permeability [16], [17], cardiotoxicity [18], [19], metabolic stability [4], [20], and solubility [21] have been developed to accelerate drug discovery. Recently, there have been numerous advancements in message-passing methods that are trained to predict molecular properties [22], [23], [24]. Such graph networks use dense layers of neural networks as non-linear functions for message passing convolution and are also commonly known as graph-convolutional neural networks (GCNNs). Graph-based models are naturally well suited for molecular modeling because atoms may be modeled as nodes and bonds as edges in mathematical graphs to represent molecules. The main advantage of GCNNs is that they consider more distant information through iterative message-passing operations, avoiding the local dependencies of descriptor-based models, such as molecular fingerprints. GCNNs have outperformed previous descriptor-based ML approaches in various molecular property prediction tasks [25], [26], [27], [28]. In addition, various attempts have been made to combine the attention mechanism module with GCNNs to increase prediction performance by capturing global dependencies between functions in substructures [29], [30], [31], [32]. The advent of AI-based drug discovery platforms marks a transformative moment in medical research, significantly influencing public health and society at large. These platforms accelerate the discovery process, enabling the rapid identification of potential therapeutic candidates with suitable pharmacokinetic properties, and reducing the time and cost involved in traditional methods of drug discovery. Consequently, this has the potential to revolutionize healthcare by making novel and effective treatments more readily available, especially in underserved or resource-limited settings.

**Fig. 1:**
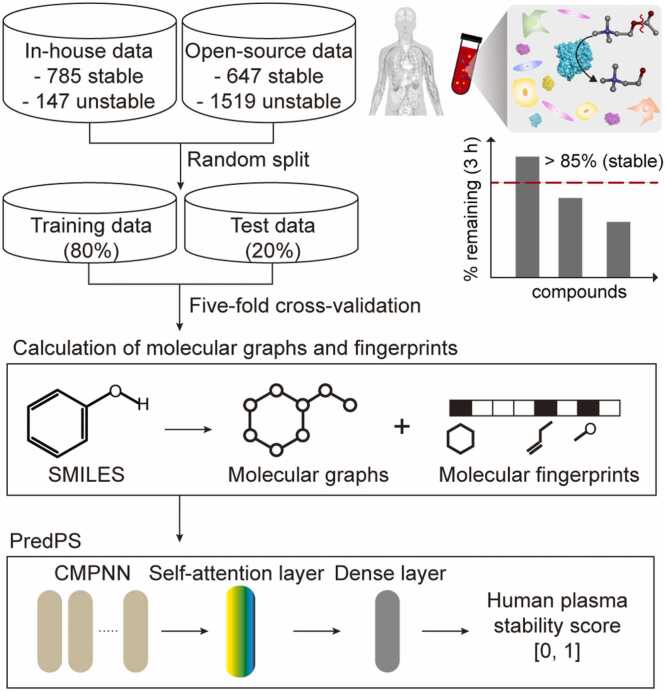
Relation schematic workflow of PredPS. PredPS predicts the human plasma stability for a given compound as a binary classification—stable or unstable. PredPS is based on an attention-based message passing neural network trained with in-house and open-source data, comprising a chemically diverse set of chemical compounds.

In this study, we developed an attention-based graph neural network called PredPS, which predicts the plasma stability of a given compound in human plasma and classifies the compound as stable or unstable (Fig. 1). We first generated in-house data on the plasma stability of a diverse set of 932 compounds using an in vitro assay in human plasma (785 stable and 147 unstable compounds) to develop PredPS. In addition, we collected open-source data on 2166 compounds (647 stable and 1519 unstable compounds) for human plasma stability. We then constructed an attention-based graph neural network (PredPS) to predict human plasma stability. Predicted results for human plasma stability are returned as a binary classification—stable or unstable. To evaluate the performance of PredPS, we also tested four ML and DL algorithms: random forest (RF), support vector machine (SVM), directed message passing neural network (DMPNN) [25], and communicative message passing neural network (CMPNN) [28]. PredPS showed the highest area under the receiver operating characteristic curve (AUC) of 0.901 ± 0.006 when evaluated using 5-fold cross-validation. To the best of our knowledge, this is the first time that a model based on deep learning has been used to predict human plasma stability. Our model can be used for binary class prediction of a compound and high-throughput screening of chemical compounds in the early stages of drug discovery.

## 2 Materials and methods

### 2.1 In vitro human plasma stability assay

All compounds used in the human plasma stability assay were obtained from the Korea Research Institute of Chemical Technology (Daejeon, Korea). Pooled plasma was purchased from Innovative Research, Inc. (Novi, MI, USA). Test compounds were spiked into the preincubated 100% plasma (pH 7.4) to produce a final compound concentration of 2 µM with a final DMSO concentration of 2%. The spiked plasma samples were incubated at 37 °C, and the reactions were terminated by adding a sufficient volume of acetonitrile containing disopyramide as an internal standard. The compound concentrations in the supernatant were analysed by LC-MS/MS after centrifugation at a relative centrifugal force of 3220 g for 20 min at 4 °C.

### 2.2 Open-source human plasma stability

We collected human plasma stability data from public databases—PubChem [33] and ChEMBL [34]—for model training. The information collected provides two types of human plasma stability data: the concentration (%) of the compound remaining after a certain time and the half-life (t_1/2_).

### 2.3 Data preparation

We first standardized the simplified molecular-input line-entry system (SMILES) format of all collected compounds using RDKit (http://www.rdkit.org) and MolVS (https://github.com/mcs07/MolVS) after collecting in-house and open-source datasets. The standardization process included the selection of the largest fragment, removal of explicit hydrogens, ionization, and calculation of stereochemistry.

For the in-house dataset, compounds with ≥ 85% remaining after 3 h in human plasma were considered stable structures, whereas compounds with < 85% remaining were considered unstable structures [1], [35]. For the open-source dataset, compounds were considered stable if at least 85% of the compound remained in human plasma after 3 h. Assuming that the compound decreased linearly by 85% in plasma within 3 h, the half-life was approximately 10 h. Therefore, compounds with a half-life of ≥ 10 h in human plasma were considered stable, and compounds with a half-life of < 10 h were classified as unstable.

### 2.4 Baselines

We used RF, SVM, DMPNN [25], and CMPNN [28] as four baseline methods to compare our PredPS with traditional ML methods and existing graph convolution networks. RF is a supervised learning algorithm with an ensemble of decision trees generated from a bootstrapped sampling of features. It is regarded as the gold standard in structure-property relationship research owing to its robustness, ease of application, and high prediction accuracy [4], [36], [37]. The SVM method was proposed by Vapnik and is based on the structural risk minimization principle [38]. An estimated function is a linear extension of a function defined over a particular collection of data (support vectors). The input data were mapped onto a high-dimensional feature space, and linear regression was performed in the feature space. The extended connectivity fingerprint with a fixed length of 1024 was used with the RF model and SVM, which was implemented in Python 3.6.13, with the Scikit-learn package, version 0.24.2 [39]. For the RF model, we set 500 trees suggested in metabolic stability [4]. An SVM model with a radial basis function kernel was used for plasma stability. Both RF and SVM were evaluated using 5-fold cross-validation.

Recently, structural information of compounds has been encoded by MPNN [40], which is widely used to predict molecular properties. MPNN refers to a method of continuously updating node information corresponding to atoms when a molecular structure is expressed as a graph in graph convolution. In this study, we employed the DMPNN and CMPNN, which are MPNN variants, as graph-based baseline methods. MPNN focusses primarily on achieving node (atom) embeddings while ignoring information carried by edges (bonds). A DMPNN uses messages involving directed edges (bonds) [25] to compensate for this problem. For the central node of the graph, the information of the central node is updated by mixing the edge information from neighboring nodes connected by intermolecular bonds. Information is transmitted according to the surrounding environment of each node and the structural features of the molecules can be effectively encoded by repeating this process several times. The CMPNN was developed to improve the insufficient representation of the attribution of molecular graphs in the DMPNN [28]. This method reinforces the node-edge interactions using the ‘communicative’ kernel. The DMPNN was implemented with the source code obtained from ChemProp (https://github.com/chemprop/chemprop), and the CMPNN was implemented with the source code obtained from https://github.com/SY575/CMPNN.

### 2.5 Model architecture and training of PredPS

**Fig. 2:**
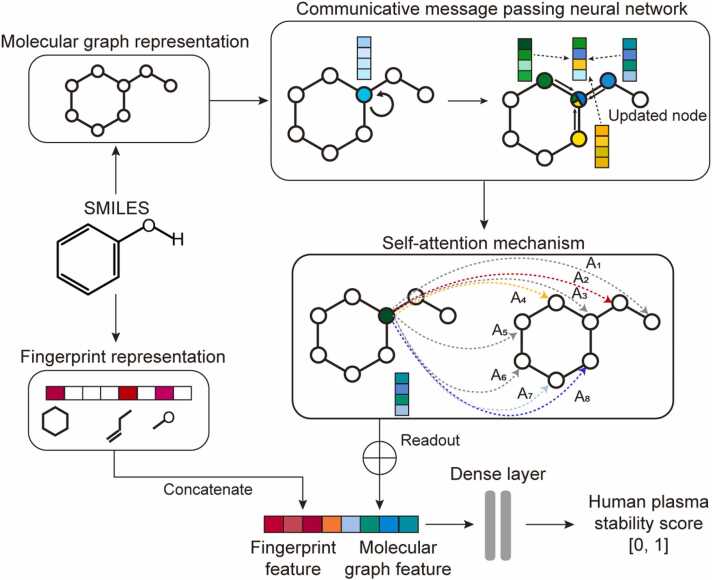
Model architecture of PredPS. PredPS integrates the communicative message passing neural network (CMPNN) [28], a self-attention layer, and fully-connected layers. It transforms input compounds (SMILES) into molecular fingerprints and graph representations, updating all node and edge features. The self-attention method generates a molecular feature vector, which when concatenated with graph features, undergoes training as fully connected layers.

PredPS comprises a CMPNN encoder, a self-attention layer, and fully connected layers optimized over a molecular fingerprint representation concatenated with a graph-based representation (Fig. 2). We considered two types of molecular representations: molecular fingerprints and graphs. For molecular fingerprints, we used the Morgan fingerprint [41] provided by the Python package RDKit (http://www.rdkit.org) to convert the SMILES strings into binary feature vectors of 2048 bits. For molecular graphs, all node and edge features were initialized using the atom and bond properties, respectively (Supplementary Table 1). For all nodes in the graph, a node message vector is updated by an aggregate function based on the message booster [28] using the former hidden states of all neighboring nodes. The hidden state of each node is updated by a communication function using the message vector and former hidden state. We adopted a multilayer perceptron as the communicate function showing the best performance benchmarked by Song et al. [28]. Subsequently, the edge message vector is updated by subtracting the former hidden state of the inverse bond from the hidden state of the node. Then, the hidden state of the edge is updated by feeding it into a fully connected layer with its initial hidden state as the bias and ReLU activation function. These procedures update the hidden states of nodes and edges five times, and the final message vector and hidden state vector are calculated using the aggregate and communicate functions, respectively. Next, the self-attention method was applied to the readout procedure for all nodes to generate a molecular feature vector [42]. Finally, we concatenated the representation from molecular graphs to the molecular fingerprints and trained fully connected layers to predict human plasma stability as a binary classification of stability or instability. More detailed embedding information and hyperparameters are provided in Supplementary Table S1, S2, and Fig. S1.

## 3 Results and discussion

### 3.1 Preparation of in-house and open-source dataset for human plasma stability

**Fig. 3:**
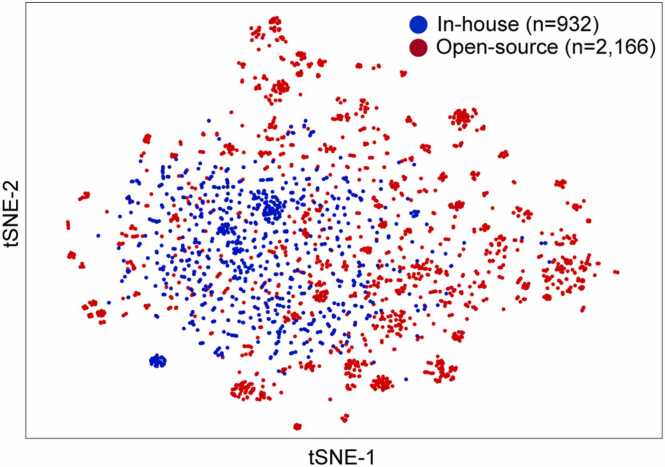
Visualization results of chemical diversity using t-distributed stochastic neighbor embedding (t-SNE). Blue indicates in-house data, and red indicates open-source data for human plasma stability.

Building generalizable and robust deep-learning models requires a sufficient amount of input data with various unbiased characteristics. High-quality input data were prepared by integrating in-house datasets obtained from in vitro human plasma stability measurements with human plasma stability datasets from public databases. Particularly, in the case of the in-house dataset, the quality of the dataset was determined to be excellent by measuring the human plasma stability of compounds with various scaffolds from the Korea Chemical Bank (www.chembank.org) under consistent assay conditions. High-dimensional data were projected onto a low-dimensional space using t-distributed stochastic neighbor embedding (t-SNE), a dimensionality reduction method, to investigate the diversity of molecular properties. Molecular representations based on Morgan fingerprints were used as inputs for t-SNE for 3098 compounds (Fig. 3).

As shown in Fig. 3, compounds in the open-source dataset appeared clustered, indicating that several compounds had similar molecular properties. In contrast, the compounds in the in-house dataset were relatively more evenly distributed on t-SNE. Furthermore, the plasma stability data of the open-source dataset mainly showed a high proportion of unstable compounds, such as pro-drugs. In contrast, the proportion of stable compounds in the in-house dataset is high. Training data with low chemical diversity or class imbalance can cause overfitting and model generalization problems [43]. We integrated in-house and open-source datasets to prepare training datasets with various compound structures to avoid these problems. Simultaneously, the ratio of stable/unstable compounds was similarly prepared.

PredPS was specifically designed and trained on a diverse dataset consisting primarily of small molecules. As a result, the program shows strong performance when applied to small molecules. As such, there are potential limitations when extrapolating this model to more complex molecular structures, such as cyclic peptides. Macrocyclic or bicyclic peptides may not be optimally predicted by PredPS due to their structural complexity and the lack of these compounds in the training set. The application of PredPS to these types of molecules is an exciting future prospect, but researchers should consider that accuracy may be lower than reported for small molecules.

### 3.2 Evaluation of prediction performance of PredPS

**Fig. 4:**
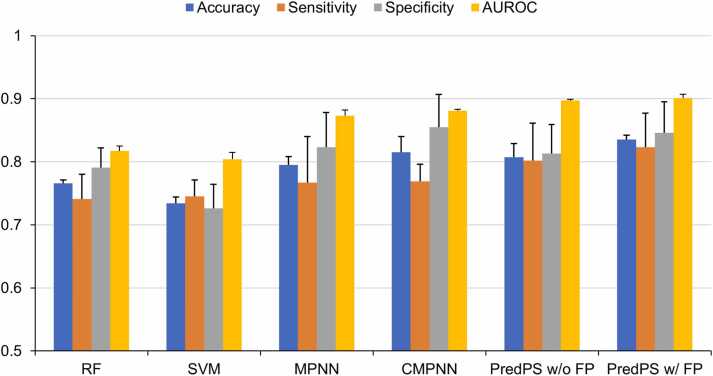
Performance results of PredPS and existing molecular representation methods for the plasma stability. AUROC, area under the receiver operating characteristic curve; RF, random forest; SVM, support vector machine; MPNN, message passing neural network; CMPNN, communicative message passing neural network; FP, fingerprint.

PredPS achieved an overall accuracy of 0.835 ± 0.007, AUC of 0.901 ± 0.006, sensitivity of 0.823 ± 0.054, and specificity of 0.846 ± 0.049 in the 5-fold cross-validation (Fig. 4 and Table 1). PredPS outperforms traditional ML models (RF and SVM) and existing graph-based neural networks (MPNN and CMPNN). The PredPS was constructed by connecting the self-attention layer to the CMPNN model architecture. An attention layer was applied to capture the importance of substructures in determining plasma stability instead of simply combining all the learned representations with sum pooling after the message-passing neural encoder. We confirmed that PredPS using attention pooling had a higher AUC value than CMPNN alone. Furthermore, all performance metrics improved when the fingerprint features obtained using the Morgan algorithm [41] were concatenated with the final graph representation (Fig. 4 and Table 1). Molecular fingerprints can provide explicit structural information by capturing properties related to molecular substructures, including aromatic rings and functional groups.

**Table 1:** Performance results of PredPS and existing molecular representation methods on the internal dataset.

	RF	SVM	DMPNN	CMPNN	PredPS w/o FP	PredPS w/ FP
Accuracy	0.766 ± 0.005	0.734 ± 0.010	0.795 ± 0.013	0.815 ± 0.025	0.807 ± 0.022	0.835 ± 0.007
Sensitivity	0.741 ± 0.039	0.745 ± 0.026	0.767 ± 0.073	0.769 ± 0.027	0.802 ± 0.059	0.823 ± 0.054
Specificity	0.791 ± 0.031	0.726 ± 0.038	0.823 ± 0.055	0.855 ± 0.052	0.813 ± 0.046	0.846 ± 0.049
AUC	0.817 ± 0.008	0.804 ± 0.011	0.873 ± 0.009	0.881 ± 0.002	0.897 ± 0.002	0.901 ± 0.006

Random forest (RF) and support vector machine (SVM) were implemented using Scikit-learn package. The DMPNN was implemented with the source code obtained from ChemProp (https://github.com/chemprop/chemprop), and the CMPNN was implemented with the source code obtained from https://github.com/SY575/CMPNN. We employed a 5-fold cross-validation with a random split and provided the mean and standard deviation for each performance metric.

Sensitivity (recall rates) was computed to estimate the risks of false negatives because it was more severe to predict an actual unstable compound as stable. False negatives can mislead medicinal chemists to continue working futilely on unstable compounds, wasting time and resources. The sensitivity of the test set was 82.3%. A higher sensitivity score indicated a lower risk of false negatives. The high AUC and sensitivity values indicate that PredPS shows high accuracy in predicting human plasma stability, sufficient for ADMET screening in the early stages of drug discovery.

### 3.3 Attention analysis

**Fig. 5:**
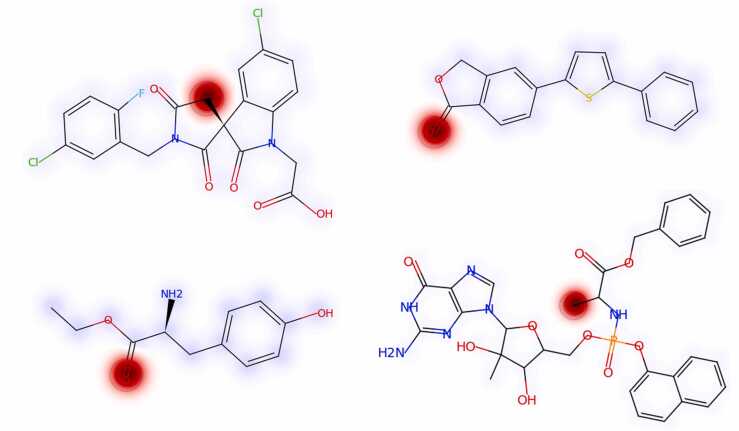
The color of molecules on a heat map depends on the plasma stability. Red represents a predicted unstable feature, while blue represents a predicted stable feature.

For PredPS, attention weight scores from the self-attention mechanism were obtained to identify learned features. We examined the attention patterns to assess whether the model focused on particular molecular substructures to predict plasma stability. Six unstable chemicals from the training set were randomly selected to analyze attention patterns. The most unstable compounds in plasma have ester bonds [1]. Visualizing the attention weight scores confirmed that the model focused locally on the atoms constituting the ester bond, which was consistent with expectations (Fig. 5).

### 3.4 Basic usage of web based PredPS

**Fig. 6:**
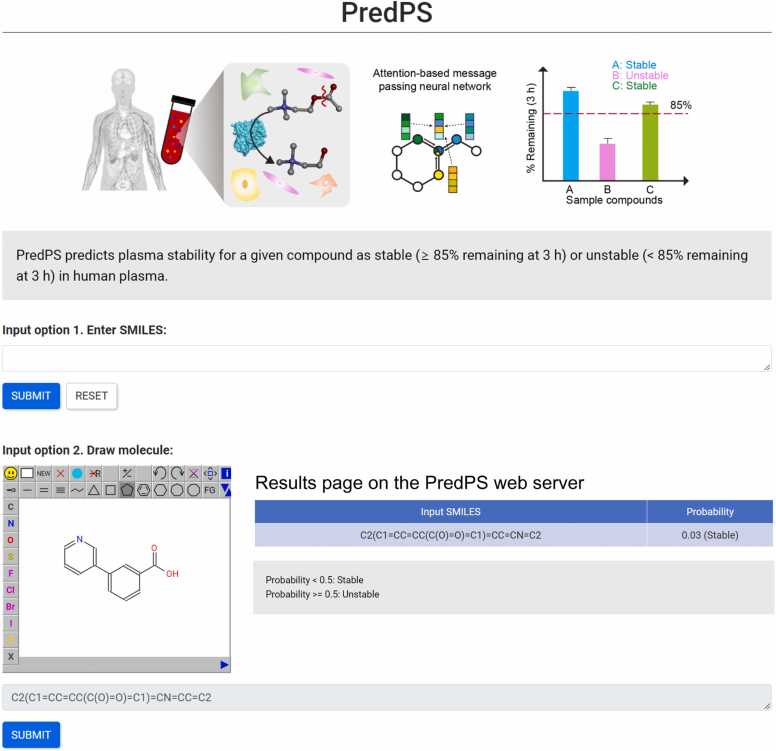
User interface of PredPS web server (https://predps.netlify.app). Input data can be obtained by directly entering SMILES or drawing the target chemical. Stability predictions for human plasma are either stable or unstable as binary outcomes.

A publicly accessible web server was created to predict the human plasma stability of the requested compound. It accepts the SMILES format of the query compound as the input and returns binary classification results as stable or unstable. The user interface of the web server is illustrated in Fig. 6. Users can also directly draw the chemical structure of a query compound to predict plasma stability.

AI-based plasma stability prediction platforms can be widely utilized in the pharmaceutical industry. By being able to screen only compounds with good plasma stability in the early stages of drug discovery, the organic synthesis process and preclinical stages of compounds can be streamlined. As a result, these platforms can accelerate the drug discovery process, quickly identifying potential therapeutic candidates and reducing the time and costs associated with traditional drug discovery methods.

## 4 Conclusion

In this study, we proposed a plasma stability prediction tool, PredPS, which classifies input compounds as stable and unstable in human plasma. The PredPS comprises a CMPNN encoder and a self-attention layer. PredPS showed the highest accuracy, sensitivity, and AUC based on a comparative analysis using traditional ML methods and existing graph-based neural networks. In addition, we developed a publicly accessible web server to predict the stability of the human plasma. Although the evaluation of plasma stability is very important in drug development, there are no known open-source programs that predict the stability of compounds in human plasma. PredPS could serve as a helpful tool for predicting the human plasma stability of compounds in the early stages of drug discovery and development. In particular, using an in silico-based plasma stability prediction model in the high-throughput screening step is a very effective way to save time and money. The emergence of AI-powered drug discovery platforms represents a transformative moment in medical research, with major implications for public health and society at large. As a result, this has the potential to revolutionize healthcare by making new and effective treatments more readily available, especially in underserved or resource-limited settings.

## CRediT authorship contribution statement

W.D.J. and K.-S.O. designed research; W.D.J. contributed model developments; J.S.S. and S.A. performed in vitro assays. W.D.J., J.J., and K.-S.O. analyzed data; W.D.J., J.J., and K.-S.O. wrote the paper.

## Declaration of Competing Interest

The authors declare that they have no known competing financial interests or personal relationships that could have appeared to influence the work reported in this paper.

## Data Availability

The source code for PredPS is available at https://bitbucket.org/krict-ai/predps including an example data set and the PredPS web server is available at https://predps.netlify.app.
