# Supplementary material for: PredPS: Attention-based graph neural network for predicting stability of compounds in human plasma
Source: Comput Struct Biotechnol J. 2023 Jul 7;21:3532–9. doi: 10.1016/j.csbj.2023.07.008 (PMC10362732; doi:10.1016/j.csbj.2023.07.008)
Supplement: Supplementary file 1 — Supplementary material. [file mmc1.docx]

**Supplementary data**

PredPS: Attention-based graph neural network for predicting stability of compounds in human plasma

Woo Dae Jang^a,*^, Jidon Jang^a^, Jin Sook Song^a^, Sunjoo Ahn^a,b^, Kwang-Seok Oh^a,b,*^

^a^*Data Convergence Drug Research Center, Korea Research Institute of Chemical Technology, Daejeon 34114, Republic of Korea*

^b^*Department of Medicinal and Pharmaceutical Chemistry, University of Science and Technology, Daejeon 34129, Republic of Korea*

⁎ Corresponding authors.

*E-mail addresses*: wdjang@krict.re.kr (W.D. Jang), ksoh@krict.re.kr (K.-S. Oh).

## **Supplementary Figure**


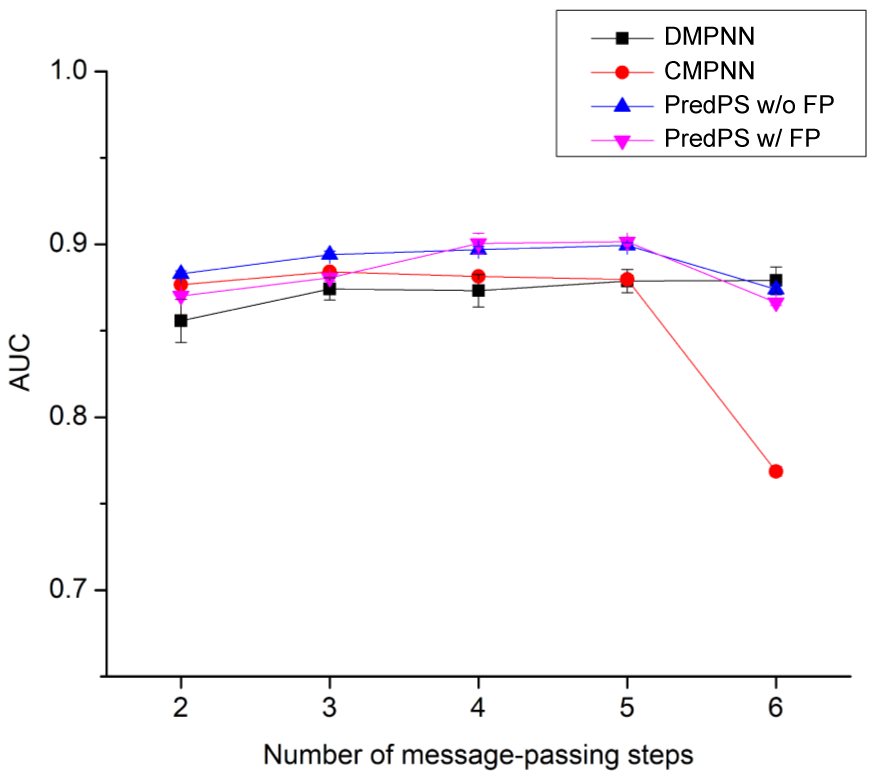


**Fig. S1.** AUC values according to depth of message-passing neural networks.

## **Supplementary Tables**

**Table S1.** **Description of Atom and Bond Features for PredPS*^a^***

| **Attribute** | **Description** | **Dimension** |
| --- | --- | --- |
| Node (atom features) | | |
| Atom type | Type of atom (e.g., C, N, O, S, F, Si) by atomic number | 100 |
| Degree | Number of heavy atom neighbors | 6 |
| Formal charge | Charge assigned to an atom (− 2, − 1, 0, 1, 2) | 5 |
| Chirality label | Unspecified, tetrahedral CW/CCW, or other | 4 |
| # of hydrogens | Number of bonded hydrogen atoms | 5 |
| Hybridization | sp, sp^2^, sp^3^, sp^3^d, or sp^3^d^2^ | 5 |
| Aromaticity | Whether the atom is in an aromatic system | 1 |
| Atomic mass | Mass of the atom, divided by 100 | 1 |
| Edge (bond features) | | |
| Bond type | Single, double, triple, or aromatic | 4 |
| Conjugated | Whether the bond is conjugated | 1 |
| Ring | Whether the bond is part of a ring | 1 |
| Bond stereo | None, any, E/Z or cis/trans | 6 |

*^a^*All features are embedded as one-hot encodings except for atomic mass.

**Table S2. Hyperparameters Optimization for PredPS*^a^***

| **Hyperparameters** | **Considered values** |
| --- | --- |
| Activation function | ReLU, LeakyReLU, PReLU, tanh, ELU, SELU |
| Message-passing steps | 2, 3, 4, 5, 6 |
| Graph embedding size | 100, 200, 300, 400, 500 |
| Learning rate | 10^-3^, 10^-4^, 10^-5^ |
| Dropout rate | 0.0, 0.05, 0.1, 0.15, 0.2, 0.25, 0.3 |
| Number of layers in fully connected network | 1, 2, 3 |

*^a^*ReLU, rectified linear unit; PReLU, parametric ReLU; tanh, hyperbolic tangent; ELU, exponential linear unit; SELU, scaled exponential linear unit.
